# Body Satisfaction, Weight Stigma, Positivity, and Happiness among Spanish Adults with Overweight and Obesity

**DOI:** 10.3390/ijerph17124186

**Published:** 2020-06-12

**Authors:** Débora Godoy-Izquierdo, Juan González-Hernández, Alejandra Rodríguez-Tadeo, Raquel Lara, Adelaida Ogallar, Estefanía Navarrón, María J. Ramírez, Clara López-Mora, Félix Arbinaga

**Affiliations:** 1 Departamento de Personalidad, Evaluación y Tratamiento Psicológico, Facultad de Psicología, Universidad de Granada, Campus Universitario de Cartuja, 18071 Granada, Spain; jgonzalez@ugr.es (J.G.-H.); adelaidaogallar@ugr.es (A.O.); 2 Grupo de Investigación Psicología de la Salud y Medicina Conductual (CTS-267), Centro de Investigación Mente, Cerebro y Comportamiento, Facultad de Psicología, Universidad de Granada, Campus Universitario de Cartuja, 18071 Granada, Spain; rlaramoreno@ugr.es (R.L.); enava@correo.ugr.es (E.N.); mariaps@correo.ugr.es (M.J.R.); claralopezmora@gmail.com (C.L.-M.); 3 Departamento Ciencias de la Salud, Instituto de Ciencias Biomédicas, Universidad Autónoma Ciudad Juárez, Anillo Envolvente del Pronaf y Estocolmo, Ciudad Juárez 32300, Chihuahua, Mexico; alrodrig@uacj.mx; 4 Departamento de Psicología Social, Facultad de Psicología, Universidad de Granada, Campus Universitario de Cartuja, 18071 Granada, Spain; 5 Seneca Foundation & Departmen of Human Development and Family Science, University of Missouri-Columbia, Gentry Hall, Columbia, MO 65211, USA; 6 Departamento de Psicología Clínica y Experimental, Facultad de Educación, Psicología y Ciencias del Deporte, Universidad de Huelva, Campus Universitario El Carmen, 21071 Huelva, Spain

**Keywords:** subjective well-being, body image, weight-related stigma, positive disposition, obesity and overweight, health promotion

## Abstract

Although previous evidence suggests that happiness is lower among individuals with obesity, research on the correlates of subjective well-being (SWB) is warranted to increase our knowledge. We aim to explore excess weight (i.e., measured and self-reported body mass index (BMI)), body image and satisfaction, self-stigma, positivity, and happiness among Spanish adults with overweight or obesity. We further aim to investigate the predictors of SWB in this sample. A convenience sample of 100 individuals with excess weight completed self-reports on the study variables and were weighed and their height measured. On average, the participants reported body perceptions revealing minor excessive weight, moderate body satisfaction, low-to-moderate weight-related stigma, and elevated positivity and happiness. BMI and gender/sex independently affected these variables, but there were no significant interaction effects. Furthermore, individuals with overweight or obesity with higher body satisfaction and elevated positivity were more likely to report being happy, independent of their age, gender/sex, weight, and weight-related stigma. Mediation effects were found for body satisfaction and positivity in the relationship between weight and happiness. Moreover, positive orientation suppressed the pervasive influence of stigma on SWB. Our findings confirm the key role of body image dimensions and weight-related stigma for happiness and add support to the relevance of positivity for overall well-being of individuals with excess weight. These results may inform obesity management actions focused on inclusive aesthetic models, combating social stigmatization and enhancing positivity for a flourishing and fulfilling life.

## 1. Introduction

Given that body image is now widely recognized as an important public health concern because of the increasing worldwide incidence of negative body perceptions and body dissatisfaction [1], it is essential to understand body image issues among individuals with excessive weight. Research on body image and body satisfaction in individuals with excess weight is extensive [2,3]. Most people with excess weight perceive themselves as a person with overweight or obesity, even when they do not have completely accurate perceptions of their weight. In addition, a positive correlation between body mass index (BMI) and body dissatisfaction has been found. A considerable number of persons with overweight and obesity are dissatisfied with their bodies and wish they were thinner [3,4,5]. Women with obesity are significantly more dissatisfied with their bodies compared to men with obesity [3,5].

Moreover, body weight is associated with psychosocial well-being in individuals with excessive weight. It has been found that people with obesity experience weight-related prejudice, discrimination, and stigma in many social areas, including education, employment and healthcare contexts, interpersonal and intimate relations, and mass media presentation [6,7]. As a consequence of weight stigma, they also experience numerous and pervasive outcomes in terms of functioning, quality of life, and well-being [8,9], including unsuccessful efforts to manage excess weight, weight gain, and obesity maintenance. Nonetheless, weight-related stigma has been scarcely investigated among Spanish adults with excess weight (e.g., [10,11]).

Shape–weight concerns, weight-related stigma, and psychological distress have been found to define in combination a psychosocial profile of experiences with obesity among individuals with excessive weight [12]. Besides psychopathological characteristics, unhealthy attitudes and behaviors, the risk for diseases and eating disorders, weight-loss failure, and diminished quality of life, research on positive states of well-being and protective factors configuring alternative psychosocial profiles is, unfortunately, scarce. 

It has been observed that happiness is diminished among people with excess weight [13,14,15,16]. It is well established that subjective well-being (SWB) shows an inverse relationship with BMI [13,14,17,18,19]. Moreover, the association between obesity and decreased happiness affects females more than males [18,19,20,21]. Research on the impacts of the obesity epidemic is essential; nonetheless, one relatively neglected topic in the literature is the relationship of obesity with SWB [13]. There are only a few studies on the happiness of Spanish individuals with obesity [10,11,22,23]. However, many individuals with obesity are happy (e.g., [24,25]), and many more pursue the highest hedonic well-being and life satisfaction as possible.

Positivity [26], a “trait-like basic disposition” characterized by a positive cognitive orientation towards self, life, and future, lies at the core of self-esteem, life satisfaction, and optimism [27]. As such, positivity has beneficial effects for optimal human functioning, achievement, and adaptation across life domains and may act as a protective factor against mental illness. It has been proposed as a dispositional component of SWB [27,28]. Positivity has not been previously studied among people living with excess weight. Thus, one of the main contributions of the present study is to offer some pioneer data on positive orientation in individuals with excess weight and the relationship between positivity and happiness when considering body satisfaction and weight-related stigma.

In sum, whereas research has been mostly focused on the harmful consequences of obesity for psychological well-being, our main aim is to contribute by exploring happiness and its correlates in individuals with excessive weight. Given that body dissatisfaction and self-stigma have been consistently related to decreased functioning and well-being, we were interested in studying the possible contribution of positive body image and positivity to SWB. Noteworthy, there is no study, as far as we know, on positivity in individuals with obesity, and there is a scarcity of research conducted with Spanish samples on self-stigma. Thus, our findings are expected to make a relevant contribution to knowledge on the correlates of obesity. Specifically, in the present study, we aim to explore excess weight (i.e., measured and self-reported BMI), body image and satisfaction, self-stigma, positivity, and happiness among Spanish adults with overweight or obesity. We further aim to investigate possible predictors of SWB among this population. We expected to find that positive body perceptions, body satisfaction, and a positive orientation would demonstrate a positive relationship with SWB, whereas self-stigma would inversely predict the participants’ happiness.

## 2. Materials and Methods

### 2.1. Participants and Procedure

A total of 100 adults from 19 to 57 years old (average age: 42.03 ± 10.74, 60% women), residing in southern Spain, voluntarily participated in the study. All participants had a BMI ≥ 25 (69% overweight: 45% overweight I, 24% overweight II; 31% obesity: 22% obesity I, 9% obesity II). No differences were found for age between women and men, and between participants with overweight and obesity either (see Table 1). All participants were white and had an average socioeconomic status (Social class was determined by unifying education level, job status, and income. Following the definition by the Health Determinants Taskforce of the Spanish Epidemiology Society, six categories were established, which were regrouped into three: high (Class I–II), middle (Class III–IV), and low social classes (Class V–VI) [29]). Recruitment was conducted with a convenient, nonprobabilistic procedure according to the inclusion criteria (i.e., having overweight or obesity, not suffering from severe physical and mental diseases, being 18–65 years old) in local medical settings.

Specifically, recruitment was conducted in two primary care consultations among individuals with obesity, who consulted on weight and health during March 2019. After inviting the individuals to voluntarily participate and after informing them about a study on well-being and health in adults with excessive weight, the anonymous nature of the data, and the research participants’ rights, we obtained their written consent. Then, assessment was conducted in a medical examination room. First, sociodemographic data and self-reported weight and height were collected in an interview format. Then, self-reported data on body perceptions, self-stigma, positivity, and happiness were collected. The order of the questionnaires was counterbalanced to avoid order biases. Finally, objective measures of weight and height were obtained.

Approval was obtained from the ethics committee of the authors’ university (CIEB-2018-1-36). The procedures used in this study adhere to the tenets of the Declaration of Helsinki of 1975, revised in 2013.

### 2.2. Study Variables and Measures

Sociodemographic data were collected from the participants. Based on self-reported weight and height, we calculated BMI as kg/m^2^. We also measured weight and height with a mobile anthropometer (Aicok Weight Scale, mod. CF398BLE, USA), which uses bioelectrical impedance analysis technology for monitoring multiple physical indexes, including body weight (weight range up to 400 pounds/180 kg and indexing value accurate to 0.2 pounds/0.1 kg) and BMI. The participants were weighed while erect, with the arms along the body, in bare feet and light clothes. BMI based on measured weight was categorized according to international standards on nutritional status in the adult population [30,31], i.e., <18.5 low weight, 18.5–24.9 normal weight, 25.0–29.9 overweight (25.0–26.9 overweight type I and 27.0–29.9 overweight type II), and ≥30.0 obesity (30.0–34.9 obesity type I, 35.0–39.9 obesity type II, and ≥40.0 obesity type III). The accuracy of body weight estimation was calculated as the discrepancy between objective and subjective BMI. For the analyses, measured BMI was used, unless any other specification is made.

The perceptual component of body image [32] was explored by using silhouettes corresponding to different BMI ranges [33]. A total of 15 male or female body figures were presented to the individuals to assess their own perceived bodies (perceived body image, PBI) and ideal body (ideal body image, IBI) (in both cases, −7 = excessively obese, 0 = excessively thin and flaccid, 7 = excessively muscular). In addition, the desire to change weight and body appearance was assessed by the PBI-IBI discrepancy (i.e., negative values indicate a desire for a slimmer and/or more muscular body; positive values indicate a desire for a heavier and/or less muscular body; a value of 0 indicates the desire to maintain the same body and appearance). This discrepancy has been widely used as an indicator of body satisfaction [33,34,35,36]. Body dissatisfaction was also assessed by a single face-valid item (“How satisfied are you with your current body weight and appearance?” 1 = extremely dissatisfied, 7 = extremely satisfied) [33]. Body satisfaction is considered a key dimension in the evaluative–subjective component of body image [32].

Stigma and self-stigma associated with excess weight were assessed with the Spanish version [37] of the 11-item Weight Bias Internalization Scale (M-WBIS) [38]. Weight-related stigma is assessed with respect to personal competence and self-worth, attractiveness, judgments by others, desire to change weight, weight-related distress, sexual opportunities, and so forth (1 = completely disagree, 7 = completely agree). Higher scores indicate greater self-stigma (two items are reverse-scored). Cronbach’s α was 0.83 in the present study.

Positive functioning was assessed with the Spanish version [39] of the 8-item Positivity Scale [40]. Positivity is defined as the tendency to view life and personal experiences with a constructive perspective, i.e., life satisfaction, personal confidence, self-pride, hope and enthusiasm for future, social support, and so forth (1 = completely disagree, 5 = completely agree). A global score was obtained by adding response values and then dividing by the number of items, with higher scores indicating greater positivity (one item is reverse-scored). Cronbach’s α was 0.87 in the present study.

SWB was self-reported with the Happiness Scale [41]. Only the single-item indicator of current happiness (“How happy are you at the present, i.e., the last few days or weeks?” 0 = extremely unhappy, 10 = extremely happy) was used. Single-item indicators of happiness are usually used in national surveys and individual research [42]. Instead of specific indicators of satisfaction with life or hedonic balance (i.e., positive and negative affect) [43], we measured SWB at a “molar” level by assessing an individual’s summary assessment of their subjective happiness as a more global psychological phenomenon [1].

### 2.3. Statistical Analyses

Statistical analyses for the current study were conducted using SPSS 25.0 (SPSS Inc., Chicago, IL, USA, 2017). The nature and adequacy of the data were checked, and parametric assumptions were confirmed. In addition to descriptive analysis (mean and standard deviation (M ± SD) for continuous variables, n and % for categorical variables), we conducted Student’s *t*-tests for comparisons of independent samples in order to explore differences in the study variables due to gender/sex (the term “gender/sex” is used herein to emphasize the interrelation and intersectionality between the concepts, and the personal experiences, of both sex and gender) and BMI category (i.e., overweight and obesity). A correction was adopted for unequal variances of subgroups when appropriate. Cohen’s *d* effect size [44] was calculated (for equal sample sizes: *d* = |m_1_ − m_2_|/√|(s_1_^2^ + s_2_^2^)/2|; for unequal sample sizes: *d* = |m_1_ − m_2_|/√|((n_1_ − 1)s_1_^2^ + (n_2_ − 1)s_2_^2^)/(n_1_ + n_2_ − 2)|; m = mean, s = standard deviation), with *d* < 0.2 indicating a low effect size, 0.5 indicating a medium effect size, and >0.8 indicating a high effect size. Two-way analyses of variance (ANOVAs) were run to test the effect of the interaction of BMI category and gender/sex on the study variables, with a calculation of main simple effects [45]. Eta^2^ was obtained for indicating effect sizes. Furthermore, bivariate, zero-order Pearson’s correlations among the study variables were calculated (BMI as a continuous variable). Finally, hierarchical multiple linear regression analysis was used to examine the predictive validity of body image dimensions, self-stigma, and positivity on happiness, controlling for age, gender/sex, and BMI (as a continuous variable). 

## 3. Results

### 3.1. Descriptive Findings and Comparisons by Sex/Gender and BMI Category

On average, the participants perceived their current bodies to be similar to overweight, non-obese figures, and they desired a slimmer, nonmuscular body, indicating, on average, an underestimation of their real weight (i.e., the discrepancy between objective and subjective BMI; Table 1). Their body satisfaction was moderate-to-high. The participants also reported, on average, low self-stigma, high positivity, and high happiness. Compared with men, women reported bodies containing higher fat mass and a thinner ideal appearance, as well as lower body satisfaction and higher weight-related self-stigma (Table 1). Men and women did not differ in terms of a desire to change their weight (i.e., PBI-IBI discrepancy), positivity, and SWB. Participants with obesity reported a significantly more negative PBI and a less exigent IBI, corresponding to bodies with overweight; they also reported a greater desire to change their weight compared to participants with overweight. In addition, individuals with obesity showed significantly lower body satisfaction, marginally higher self-stigma, and significantly lower happiness. No differences in positivity were found between individuals with obesity or with overweight.

### 3.2. Interaction Effects of Sex/Gender and BMI Category

Two-way ANOVAs were conducted to explore the effect of the interaction of the measured BMI category and gender/sex on body satisfaction, stigma, positivity, and happiness. The interaction did not have a significant effect on body satisfaction (*F* = 0.232, *p* = 0.631), stigma (*F* = 0.187, *p* = 0.667), positivity (*F* = 0.014, *p* = 0.907), or happiness (*F* = 3.227, *p* = 0.076). For both males and females, a higher BMI was associated with lower body satisfaction, higher self-stigma, lower positivity, and decreased happiness. The interaction effect was close to significance in the case of happiness; appealing differences were observed between males and females: while there was a slight decrease in happiness as BMI increased in men (7.5 vs. 7.3 units for overweight and obesity, respectively), the difference was 1.6 units between women with overweight (7.9 units) and those with obesity (6.3 units).

### 3.3. Correlations and Hierarchical Multiple Regression for Predicting SWB

Table 2 shows zero-order, bivariate correlations among the study variables. 

We used hierarchical multiple regression to examine the effect of body satisfaction, self-stigma, and positivity on SWB while controlling for measured BMI, age, and gender/sex. Demographic variables were entered in Step 1, body satisfaction was entered in Step 2, self-stigma was entered in Step 3, and positivity was entered in Step 4. Demographic and anthropometric variables accounted for 4% of the variance in Step 1 of the regression involving happiness (corr. R^2^ = 0.04, *F* = 4.888, *p* = 0.029); neither age nor gender/sex significantly contributed to the explained variance, and only BMI was a significant predictor (*b* = −0.22, *p* = 0.029). Hence, as BMI increases, individuals are less likely to report being happy. BMI was no longer a significant predictor after body satisfaction (*b* = 0.42, *p* = 0.000) was entered in Step 2 (corr. R^2^ = 0.18, *F* = 11.745, *p* = 0.000). When self-stigma was entered into the equation in Step 3, it produced a significant increment in explained variance (corr. R^2^ = 0.24, *F* = 11.332, *p* = 0.000), and body satisfaction (*b* = 0.29, *p* = 0.006) remained a significant predictor along with stigma (*b* = −0.29, *p* = 0.004). The final model (Table 3) accounted for 33% of the variance in happiness (corr. R^2^ = 0.33, *F* = 13.099, *p* = 0.000), with body satisfaction (*b* = 0.25, *p* = 0.012) and positivity (*b* = 0.34, *p* = 0.000) as significant independent predictors of happiness. Thus, individuals with overweight and obesity with higher positivity traits and body satisfaction are more likely to be happier. It should be noted, however, that the zero-order correlation between self-stigma and happiness was significant, and it was a significant predictor in Step 3, implying that the finding in Step 4 might be a suppressor effect of positivity on self-stigma [46]. Thus, the findings provide support for the idea that the effect of BMI on happiness is mediated by the addition of a subjective experience of BMI (i.e., body satisfaction) and positivity disposition, while positivity suppresses the pervasive effects of self-stigma on SWB.

## 4. Discussion

In the present study, we explore the experiences of excess weight (i.e., BMI), body image perceptual and evaluative–subjective dimensions, self-stigma, positivity, and happiness in adults with overweight or obesity, as well as the associations among these variables.

The body perceptions reported by the participants of the present study are in line with other findings on individuals with overweight in our nation [33,34,35,36]. Compared with men, women reported significantly more negative body self-perceptions, i.e., bodies containing higher fat mass, and a slimmer ideal appearance, as well as lower body satisfaction [34,35,36]. Compared with participants with overweight, participants with obesity reported lower body satisfaction, a significantly more negative PBI and an IBI that did not differ considerably from their own body, i.e., they chose overweight bodies [34,35,36]. The interaction between BMI and gender/sex did not have an effect on body perceptions and satisfaction. For males and females, body dissatisfaction increases as BMI increases by desiring smaller body sizes [35]. While the desire to change their body (i.e., PBI-IBI discrepancy) did not vary due to gender/sex [36], individuals with obesity reported a stronger desire for change to reach their ideal body [34]. In addition, our findings are in accordance with others showing that women of all ages value and compare themselves to a thin, lightly toned body while men value slender and muscular, athletic-type bodies [32]; this is also true for women and men with excessive weight [3].

Self-stigma was low-to-moderate in this sample, paralleling previous findings on perceived personal discrimination among Spanish individuals with obesity [10,11,22,23,47]. Women demonstrated higher weight-related self-stigma compared to men; as others have indicated, women are more affected by weight stigmatization [7] and group discrimination [23], yet this finding has not been previously found among Spanish individuals with obesity seeking or undergoing treatment for weight loss [10,11,23,47]. While others have not found an influence of BMI on stigma among individuals with excess weight [10,11,23], some research exists on the positive relationship between weight and weight self-stigma [47], and we found that participants with obesity showed marginally higher self-stigma compared to individuals with overweight. In the present study, the interaction between gender/sex and BMI did not have an effect on weight-related stigma.

Independent of gender/sex and BMI, the participants showed elevated positivity. As far as we know, there is no previous research on the relationship between weight, body image, and positivity, and studies on positive orientation among individuals with obesity are lacking as well. Thus, our findings on positivity are novel in body image or obesity research. Furthermore, research conducted with Spanish samples on the positivity trait is scarce, which is another contribution of this study. Our findings on participants’ positivity are comparable to those found with heterogeneous Spanish samples of university students [40] and adults not affected by severe illnesses [48], although in another study, adults from the general population demonstrated higher positivity compared to our participants with obesity [49]. Moreover, others found no differences in positivity between women and men [39,40,49]. The “positive cognitive triad” on the self, life, and future is an important variable for well-being, as it makes people capable of facing life in spite of adversity, failure, loss, or serious illness [27]. In Spanish samples, it has been demonstrated to be different among individuals being treated for adjustment disorder or cardiovascular disease compared to the general population; positivity is capable of predicting the individuals’ illness condition and is associated with adaptive coping and self-efficacy for negative emotion regulation [48,49]. It also may help individuals to manage their health more successfully, as patients with cardiovascular disease participating in a rehab program demonstrated higher positivity than healthy controls [49]. Thus, we encourage researchers to deeply explore positivity and its outcomes regarding weight control, appearance management, stigma influence, and overall well-being of people living with excessive weight.

Participants in the present study reported average happiness that mirrors data from the latest EU Eurostat [https://ec.europa.eu/eurostat/] and the Spanish Sociologic Research Center [http://www.cis.es/], although the levels in this study were 1 point higher compared to the UN Gallop World Pull [50]. Others have found that Spanish individuals with obesity are, on average, happy [11], whereas other findings have supported decreased SWB for them [10,22]. Participants with obesity demonstrated significantly lower SWB compared to individuals with overweight [11]. Moreover, although no gender/sex differences were established for SWB, different trends were observed between males and females for happiness when BMI was considered: happiness is less impacted by BMI increases among men, but women feel considerably unhappier if they get fatter [18,21]. Given that the female participants in this study also demonstrated lower body satisfaction and higher weight stigma compared to male participants, this finding might be derived from the higher relevance imposed on the feminine body and the internalization of the beauty standards compared to males [32]. Nevertheless, some authors have not found effects of age, sex, and BMI on psychological well-being (i.e., self-stigma, happiness) among individuals with obesity [10,11,22,23].

In the present study, participants with lower BMI, higher body satisfaction, suffering from lower stigmatization phenomena, and holding more positive expectations about the self, life, and future were also more likely to report feeling happier. We also found that body satisfaction and positivity were independent predictors of happiness when controlling for age, gender/sex and BMI, suggesting that those individuals with overweight and obesity with better body acceptance and enjoyment and with greater positive expectations about the self, life, and future were more likely to report that they were happy. The estimates shown in Table 3 seem to suggest that there is no direct effect of obesity on well-being. In contrast, the impact of objective weight on SWB was fully mediated by body satisfaction and positivity. Furthermore, the effect of subjective appreciation of weight and body on happiness persists even after controlling for BMI and when weight stigma and positivity were introduced as further regressors, implying that personal body valorisation is associated with some direct psychosocial effects on well-being. Moreover, the progressive attenuation of the coefficients from Models 1 to 4, representing the effects of BMI, body satisfaction, and stigma on happiness, suggests that the obesity effect is partially explained by the relationship between these psychosocial characteristics and positivity. 

Notably, our findings stress that without the contribution of psychological mediators in the relationship between BMI and happiness, it is hardly understandable, if detectable. Our findings further reveal that women and men demonstrate the same pattern and that the same mediators are needed to fully capture the association between BMI and happiness. Nevertheless, while the combination of these variables accounted for a significant proportion of the variance in happiness, two-thirds of the variance remains unexplained, and an avenue for future research is to investigate other contributors to happiness among individuals with obesity.

In addition, we found that participants with obesity reported lower happiness than participants with overweight, but BMI was not a significant predictor of SWB when other psychosocial variables were included in the model. It has been found that BMI is related to decreased happiness only among individuals with obesity, not among individuals with overweight and normal weight [13,17]. Furthermore, conflicting results have been obtained with perceived weight instead of BMI and the congruency between both indicators [51]. Indeed, BMI alone is not considered to be an adequate measure of obesity or a sufficient predictor of obesity-related health outcomes, as it does not fully capture the adverse effects of obesity [13]. Thus, it might be more interesting to use corporeal self-perceptions and body satisfaction rather than objective BMI-based categories [1,51,52]. Our findings support this affirmation. Thus, while evidence indicates that SWB is negatively affected by obesity after taking into account the effect of all other relevant individual-level covariates, more studies are needed to confirm this pattern, and there are still many issues to be explored regarding this relationship.

Interestingly, we found a suppression effect of positivity on self-stigma when predicting SWB, since stigma was a significant predictor before positivity was introduced to the model. Consequently, the effect of BMI on happiness was mediated by the addition of the subjective experience of BMI (i.e., body satisfaction) and the positivity disposition, and the latter suppressed the pervasive effects of self-stigma on SWB. While BMI has been linked to stigma and rejection [47], others have found that BMI is unrelated to discrimination among obesity-range individuals [10], so the exact mechanism through which stigma is linked to weight and possible protective factors against its pervasive effects should be more deeply explored in future research.

Our findings have several interesting practical derivations. Along with complete preparation of professionals in order to successfully attend obesity in its complexity [53] and actions for increasing awareness of body weight and health issues among the general public and individuals at risk or suffering from excess weight [54], public campaigns and health policies should foster the promotion of healthier, positive, and realistic body images [55] and of healthy weight-control strategies for weight and appearance management [56,57] (e.g., [58]). By listening to people’s experiences and recognizing body weight as one aspect of human diversity, we can promote inclusion and acceptance of all individuals, regardless of their body weight, shape, or size, and thus support their health, well-being, and participation throughout life. Moreover, our findings can be interpreted under the umbrella of the criticisms currently directed to the “weight-centered health paradigm” (WCHP), by which body weight is placed at the heart of the discourses about health, resulting in an enhanced adipophobicogenic environment and reduced health and quality of life of those living with excessive weight. This dominant paradigm has resulted in arguments from critical health promotion for a paradigm shift away from focusing on weight and focusing instead on health and well-being within a “healthy at every size”, more salutogenic approach [59,60]. In addition, widespread interventions among the general population, as well as specifically for the subpopulation with obesity, should focus on eradicating the ubiquitous social weight stigma and mitigating the deleterious influence of self-stigma on well-being [7]. Finally, as Caprara et al. [27] have pointed out, a recommendation for designing practices “conducive to promoting and sustaining positive orientation as a powerful driver of individuals’ flourishing” (p. 131) among individuals living with excess weight should be encouraged, given the mediating effects of positivity for both weight and weight stigma when SWB is considered.

Despite the contributions of the present study, which included examining the effects of BMI, several dimensions of body perceptions, weight stigma and, for the first time, to the best of our knowledge, positivity, on the happiness of a sample of men and women with overweight and obesity, our findings should be considered in light of several limitations. The sample is limited and probably not representative of the whole Spanish population. Moreover, the participants demonstrated, on average, moderate body satisfaction, low self-stigma, high positivity, and high happiness, possibly over-representing a “positive” subgroup of individuals with obesity or a group with positive or functional body self-perceptions. Our findings need to be replicated with broader and more heterogeneous samples throughout the nation. In addition, the relationship between BMI or body perceptions and SWB is also affected by several contextual variables, such as the prevalence of obesity and relative comparisons, social weight norms, and stigmatization social processes [19,20], as well as contextual sociopolitical and cultural factors [14]. Furthermore, other factors may have an influence on the variables explored in the present study, such as physical activity and other healthy habits, self-perceived health status, education level, employment status, or wealth/social class (e.g., [13,19,20,34,49]), but we did not explore these factors. Our findings (i.e., only one-third of the variance was explained by the model) indicate that there are likely other factors at play in explaining levels of well-being in people with obesity. Thus, future research should investigate other determinants of the relationship between anthropometrics, body appreciation, weight-related sociocultural phenomena, and happiness. Besides, we selected the measurement tools we have used, but no doubt, there are alternative instruments and approaches to gather data on the phenomena we have explored. Finally, the descriptive, correlational nature of the study limits the conclusions to be derived, and future research should adopt other research designs and analytical procedures to complement our knowledge on the issues explored herein.

## 5. Conclusions

In summary, the main findings reported in the present study provide support for the happiness of adults with overweight and obesity and the relationships of body image dimensions, weight-related stigma, positivity, and well-being (i.e., happiness) in the context of obesity. While demographics, BMI, and stigma have limited direct influence, body satisfaction and, to a greater extent, positivity accounted for significant proportions of the variance in happiness. Moreover, positive orientation suppressed the pervasive influence of stigma on SWB. The current study offers new directions for the study of SWB in obesity. Future research is required to investigate ways in which weight, body satisfaction, weight-related stigma, and positivity may interact to affect well-being and functioning in individuals with excess weight and to explore how accumulated evidence may be used to inform health interventions for preventing and managing obesity in all its dimensions.

## Figures and Tables

**Table 1:** Comparisons by sex/gender and body mass index (BMI) categories.

	Total M ± SD	Men M ± SD	Women M ± SD	*t* *p*	*d*	Overweight M ± SD	Obesity M ± SD	*t* *p*	*d*
Age	42.03 ± 10.74	43.53 ± 11.11	41.03 ± 10.46	−1.139 0.258	0.14	41.77 ± 11.05	42.61 ± 10.16	−0.362 0.718	0.08
Measured BMI	28.8 ± 3.6	29.3± 3.2	28.4 ± 3.8	−1.295 0.198	0.25	26.6 ± 1.2	33.5 ± 2.3	−15.327 ** <0.001	4.26
Self-reported BMI	27.3± 3.6	28.1 ± 2.9	26.7 ± 3.9	−1.999 * 0.048	0.40	25.3 ± 2.1	31.6 ± 2.1	−13.895 ** <0.001	3.00
Perceived body image	−1.8 ± 2.0	−1.2 ± 2.2	−2.2 ± 1.7	−2.443 * 0.017	0.52	−1.1 ± 1.8	−3.4 ± 1.4	6.094 ** <0.001	1.36
Ideal body image	0.3 ± 1.8	0.9 ± 2.1	−0.1 ± 1.5	−2.384 * 0.020	0.55	0.6 ± 1.7	−0.4 ± 2.0	2.648 ** 0.009	0.54
PBI-IBI discrepancy	−2.1 ± 1.8	−2.1 ± 2.2	−2.2 ± 1.5	−0.190 0.850	0.05	−1.8 ± 1.6	−2.9 ± 2.0	3.196 ** 0.002	0.61
Body satisfaction	4.7 ± 1.4	5.2 ± 1.1	4.4 ± 1.5	−2.815 ** 0.006	0.61	5.0 ± 1.1	4.0 ± 1.7	3.045 ** 0.004	0.70
Self-stigma	24.7 ± 11.4	21.8 ± 9.2	26.6 ± 12.3	2.208 * 0.030	0.44	23.3 ± 11.1	27.7 ± 11.4	−1.847 † 0.068	0.39
Positivity	3.9 ± 0.8	3.9 ± 0.8	3.9 ± 0.8	0.345 0.731	0.00	3.9 ± 0.8	3.8 ± 0.8	0.650 0.517	0.13
Happiness	7.5 ± 1.8	7.5 ± 1.7	7.5 ± 1.9	0.045 0.964	0.00	7.8 ± 1.6	6.8 ± 2.0	2.505 * 0.014	0.55

Note: Student’s *t*-tests for comparisons due to gender/sex and BMI category. † *p* < 0.10, * *p* < 0.05, ** *p* < 0.01.

**Table 2:** Bivariate Pearson’s correlations among the study variables.

	1	2	3	4	5	6	7	8	9
Measured BMI	1	0.92 **	−0.52 **	−0.30 **	−0.26 *	−0.38 **	0.20 *	−0.02	−0.22 *
Self-reported BMI		1	−0.49 **	−0.30 **	−0.23 *	−0.30 **	0.18 †	−0.02	−0.19 †
Perceived body image			1	0.56 **	0.52 **	0.50 **	−0.23 *	−0.05	0.10
Ideal body image				1	−0.42 **	0.28 **	−0.06	−0.11	0.01
PBI-IBI discrepancy					1	0.26 *	−0.20 *	0.05	0.10
Body satisfaction						1	−0.44 **	0.26 **	0.44 **
Self-stigma							1	−0.43 **	−0.43 **
Positivity								1	0.48 **
Happiness									1

Note. † *p* < 0.10, * *p* < 0.05, ** *p* < 0.01.

**Table 3:** Hierarchical multiple regression of happiness on body satisfaction, self-stigma, and positivity controlling for BMI, age, and gender/sex.

	Stand. Beta	Stand. Error	*t*	*p*
Measured BMI kg/m^2^	−0.08	0.045	−0.938	0.350
Body satisfaction	0.25	0.125	2.549	0.012 *
Self-stigma	−0.15	0.016	−1.541	0.127
Positivity	0.34	0.208	3.722	0.000 **

The dependent variable is happiness. Standard errors were robust to heteroskedasticity. In all the regressions, we controlled for potential confounders. * *p* < 0.05, ** *p* < 0.01.
